# How to choose an abdominal imaging fellowship

**DOI:** 10.1007/s00261-021-03170-0

**Published:** 2021-06-23

**Authors:** Derek C. Sun, Jean H. Lee

**Affiliations:** 1 Department of Radiology and Biomedical Imaging, University of California, San Francisco, 513 Parnassus Ave, S255, Box 0628, San Francisco, CA 94143-0628 USA; 2 Department of Radiology, University of Washington, Seattle, USA

**Keywords:** Fellowship, Education, Interview, Application, Abdominal Imaging, Job search

## Abstract

Radiologists in training draw from their early experiences in residency when choosing a fellowship. Once they have decided on an abdominal imaging fellowship, applicants must learn to navigate the interview process. During this challenging time, applicants explore the difference in clinical curricula and rotations, meet potential mentors and clinical faculty, consider potential academic interests and projects, and choose what location they would like to train for one year after residency. When in training, fellows undergo the challenge of finding employment while learning new skills and refining their abilities to become a well-rounded radiologist and clinician. This article summarizes key points potential applicants should consider when deciding on an abdominal imaging fellowship, how to prepare for the interview season, and how to plan their fellowship year before fellows take the next step to becoming attendings.

Choosing an abdominal imaging fellowship can be challenging. The fast-paced application season can leave undecided trainees deliberating on their future subspecialty in their second year of residency feeling rushed, lost, or left behind. Recent developments have set uniform standards for the application process while giving residents sufficient time to choose their subspecialty and begin the necessary preparations for applications and interviews [1]. Even with these changes, deciding on where to apply and settling on a program is confusing [2]. Applicants must discern stark versus subtle differences in curriculum, rotations, and clinical strengths, and faculty, potentially having a significant impact on their future careers. The purpose of this article is to help guide undecided and potential applicants to review the key factors when choosing an abdominal fellowship.

## Applications

Most fellowship program applications will require the applicant to submit a curriculum vitae (CV), 2–3 letters of recommendation, one of which may need to be provided by a residency program director, USMLE Transcripts, and a personal statement. Applicants should ask for help from other individuals to review their CVs and personal statements, as this may help improve these submissions or identify areas of confusion that may impact an applicant’s standing in the interview process.

## Interview season

The Society of Chairs of Academic Radiology Departments (SCARD) formulated a policy for fellowship interviews implementing uniformity to the interview process while giving applicants time to decide on their subspecialty [1]. Before this policy, applicants could apply and accept offers as early as their second year of radiology residency [2]. As of this writing, programs start accepting applications from third-year DR residents or applicants further in training starting August 1 with interviews beginning on November 1 and ending on March 31. These rules apply to match and non-match programs. As Abdominal Imaging Fellowships fall into a non-match category, these programs can offer positions for acceptances after interviews with a 7-day grace period for the applicant to accept or decline the offer. Once this 7-day period ends, a program can use that offer on another applicant.

While the new system gives structure to the interview process, it also compresses the interview season to the fall and early winter due to the rolling admissions process. Popular programs may fill early, and the first two weeks are critical for applicants and programs to meet each other. Smaller popular programs may fill in the first week, while larger programs may hold numerous interviews in the first week when there are a larger pool applicants as people have yet to accept positions. Applicants should prepare early and schedule their top-choice programs as the earliest interviews, preferably in the first week of the season. This ensures that there will still be available spots for offers. Waiting or scheduling interviews later in the season runs the risk of interviewing with a program that is waiting on other applicants to return an offer or run the risk of interviews being canceled as the program has already filled, increasing uncertainty in navigating the interview season. Reaching out and emailing programs to express desired interest and following up the interview with questions can be beneficial. Lastly, when deciding on a fellowship, applicants should speak to current and possibly more importantly, former fellows that have a similar desired career track, whether academics, hybrid practice, or private practice, since these alumni can give the most insight into how the program prepared them for their jobs.

## What to look for in an abdominal imaging fellowship

The purpose of a fellowship is to learn more about a subspecialty, which includes expanding core aspects of abdominal imaging and learning advanced topics to integrate these skills into one's daily practice as an expert in this growing field. However, fellowships in abdominal imaging come in different varieties. Given the recent advances in radiology techniques and patient treatments, applicants should consider which aspects they would need to focus on to round out their education. Exciting developments in imaging of deep infiltrative endometriosis, prostate cancer, hepatic evaluation, and rectal cancer have piqued significant interest from all aspects of medicine while imagers capable of reading these studies are sought out for potential employment [3].

Because abdominal imaging often overlaps with many medical subspecialties, applicants should consider programs supplying a multidisciplinary and multimodality approach to maximize their exposure. This proves beneficial when radiologists understand the impact of their interpretations on management options and patient outcomes. A common misconception among trainees is that fellowship should primarily focus on MRI (Magnetic Resonance Imaging), as residents often feel they will need more experience. While MRI plays a vital role in imaging, radiologists must understand the challenges of interpreting medical imaging across all modalities to increase their knowledge. Applicants may overlook the importance of US (Ultrasound) and CT (Computed Tomography) in evaluating diseases that complement the limitations of MRI while devoting focus towards understanding newer developments that can affect patient care. For instance, fellows should be familiar with using an iodine map on dual-energy CT to troubleshoot for active bleeding in a hematoma. And while contrast-enhanced ultrasound (CEUS) may not be implemented in every practice, fellows familiar with these techniques can offer alternative methods of diagnosing neoplasms such as renal cell carcinoma in patients unable to undergo MRI or tolerate iodinated contrast. Furthermore, fellows should be efficient in performing and confident in evaluating fluoroscopic studies ranging from upper gastrointestinal series or post-prostatectomy cystograms. And while it is imperative to have adequate MRI exposure during a fellowship, knowing that different diseases are evaluated using specific modalities and recognizing a modalities’ limitations emphasizes the educational value of having rotations on US and CT. This inclusive approach to learning abdominal radiology encourages radiologists to graduate fellowship not only with a well-rounded education but to understand the clinical impact of our interpretations and our role to patients and other doctors.

Body MRI fellowships are useful in supplementing residency training, geared towards interpreting MRI studies from the different subspecialties in radiology, including the abdomen. These programs focus portions of their educational curricula on MRI physics and understanding sequences. A dedicated fellowship in MRI can be valuable for distinct careers where a radiologist may read primarily MRI or perform MRI research, as in niche private practices or some academic or industrial settings, respectively. However, the limited exposure to other modalities may be less beneficial for fellows looking at jobs where they become a generalist in radiology. Depending on their residency training, an applicant should consider if they feel comfortable interpreting more advanced topics in abdominal imaging that may not use MRI, for example transplants and obstetrics, when deciding for an MRI-only fellowship, in addition to image-guided procedures. If a fellow is seeking to round out their education, attending an MRI-only fellowship may not necessarily help them achieve that goal. Remember that abdominal imaging fellowships can have extensive training in MRI and applicants should factor this into their decision when choosing a program.

Applicants should gear their fellowship year to not only learn new techniques but to refine their existing skills to become better radiologists. This includes recognizing intricate patterns of disease, improving efficiency of interpretations, and increasing report conciseness and clarity to convey valuable information. Fellows can use the year to improve their communication with clinicians, follow-up on pathology for correlation of radiologic diagnoses, and point clinicians towards the next steps in management or evaluation [4].

### ACGME or non-ACGME fellowships

When deciding a program, applicants have two main options: an ACGME (Accreditation Council of Graduate Medical Education)-approved fellowship or a non-ACGME-approved fellowship. Many programs in the United States are non-ACGME approved [5]. ACGME-approved abdominal imaging fellowships must adhere to guidelines listed in Program Requirements set forth by the ACGME and these programs are subject to review to renew accreditation. The educational and training curricula are structured, with requirements for faculty within the specialty, professionalism, safety, didactic teaching and conferences, and support for fellows [6]. Fellows in ACGME-approved abdominal imaging programs are based on studies applying “to diseases involving the gastrointestinal tract, genitourinary tract, and the intraperitoneal and extraperitoneal abdominal organs.” These studies are not only limited to CT and MRI but also include fluoroscopy and PET/CT. Hence, fellows in ACGME-approved programs may have a more rounded educational experience in all aspects of abdominal imaging with a more structured curriculum. In addition, these programs may have some limited elective time in other subspecialties in radiology. These programs have some lack of trainee autonomy as all fellows are under the direct supervision of faculty [6]. However, this lack of autonomy can be adjusted within the program as fellows can independently read and dictate the exams while faculty can sign the reports and give feedback to fellows afterwards, particularly later in the fellowship year.

Non-ACGME-approved fellowships may follow some similar standards to ACGME-approved programs in educational curricula and benefits, but have several distinct differences. Fellows in non-ACGME fellowships can learn and interpret studies and perform procedures in other subspecialties within radiology, including musculoskeletal and cardiothoracic radiology. Fellows can be asked to be the final interpreter of reports as part of their fellowship training, a distinct difference between ACGME and non-ACGME fellowship which can present opportunities and other unexpected benefits for post-residency training. This added flexibility presents opportunities to round out a fellow's education in preparation for their careers [7].

While this may sound daunting to residents early in their training, fellowship programs often place restrictions on the type of studies their fellows will be the final interpreter of studies as part of their clinical duties. Final signing a study can be a valuable educational experience for trainees who will soon become board-certified radiologists reading independently in their next jobs. Doing so helps radiologists still in training gauge their comfort level executing this crucial step to make the necessary adjustments to increase efficiency before graduating. Lastly, lifting this restriction ushers the opportunity for moonlighting, either at a fellow's training institution or at other hospitals, supplementing their salary during their fellowship year [7]. For most institutions, moonlighting cannot interfere with a fellows' required duties while in the program.

Fellows in ACGME-approved programs can also have moonlighting opportunities although fellows cannot be final interpreters due to ACGME policies. However, this can be a valuable experience for fellows as fellows can gradually build up their independency to prepare their careers.

### Clinical curricula

Because each abdominal imaging program has unique clinical strengths, there are factors an applicant should consider in the curricula to ensure fellows are well-prepared abdominal imagers before graduating.

The program should have a tertiary care referral center with multiple subspecialties. The institution should perform advanced techniques, including but not limited to MRI of the prostate, CT and MR urography, elastography, transplant US, obstetrics and gynecology US and MRI, thyroid US, CEUS, CT and MRI to evaluate the liver and pancreas, MRCP and secretin MRCP, rectal MRI, and image-guided procedures. Consequently, fellows should be familiar with standardized reporting systems, including LI-RADS, PI-RADS, TI-RADS, and O-RADS. Knowledge using 3-D imaging software and post-processing techniques are also emphasized during fellowship. Fellows should inquire about the different procedures they will perform, including US and/or CT-guided biopsies of the solid organs such as the thyroid, liver, or kidneys. Other fellowship programs may offer interventional procedures including drainages and ablations, while some may expand procedures to include joint, nerve, or tendon injections and other aspects of musculoskeletal ultrasound.

Abdominal emergencies are often challenging cases requiring early recognition in diagnosis for treatment. While these emergencies form the backbone curricula of most residencies, certain institutions may see a larger volume and breadth of complex cases due to referrals, including trauma, which can help supplement a fellow's education. Fellows should also consider programs that have a good diversity of emergency ultrasound to increase their familiarity with challenging cases, including ectopic pregnancies, ovarian torsion, and first-trimester pregnancies.

Applicants can inquire about subspecialized techniques that may not be available at all institutions. This includes simple procedures such as hysterosalpingography to the more advanced hysterosalpingo-contrast sonography (HyCoSy). Other studies include MR defecography or gender-affirmation imaging. A select number of fellowships offer high-risk obstetrical and fetal imaging. Jobs may require radiologists to be well-versed in specific techniques, including obstetrical imaging or other expectations including other subspecialties [3]. Besides, programs that see these subspecialized cases may come across more diverse pathology associated with these exams. Lastly, applicants should consider programs caring for various patient populations, which add to the diversity of cases, since limited access to healthcare may predispose individuals to chronic and acute conditions not seen in groups of higher socioeconomic status.

### Rotations

Fellowships rotations should include inpatient and outpatient CT, MRI, and US to ensure fellows see a wide variety of cases. Fellows should also have a procedural part in their rotations as many future jobs may require image guidance tasks to facilitate diagnosis or treatment. Applicants inquire about the number of months they are on a rotation and the number and type of studies they can expect to see on a day-to-day basis. Certain programs may have a mixed modality workflow which includes CT and MRI, while other programs may have more emphasis on specific modalities or techniques, such as a dedicated MRI rotation. While the latter can be a highly valuable and rewarding experience, applicants should decide the number of months they will be on these rotations will provide enough exposure to modality. For instance, a program that offers high-volume MRI along with CT in a cross-sectional rotation for 7–8 months of the year may be similar in the number and variety of MRI cases of a 3-month dedicated MRI rotation. For fellows who desire more time in a specific modality, programs may be flexible in trading rotations. Some future employers may require more obstetrical expertise or procedure time. Fellow may be able to ask their program director for accommodations or strike trade with their peers to accommodate such requests.

### Electives

Another important consideration in the rotation schedule is the inclusion of elective time. Fellowship programs can offer electives to help fellows supplement their training, adding flexibility to prepare for their next job and adding marketability for a potential applicant. Short stints in breast, cardiothoracic, or musculoskeletal imaging or neuroradiology can help a fellow focus on specific techniques such as needle localization, cardiac CT, or arthrograms, or reinforce learned concepts to improve speed and accuracy. Electives in interventional radiology can reacclimate trainees to procedures including biopsies, drainages, embolization, and ablations, especially if the core rotations do not have significant procedure time, although an increasing number of larger practices now defer these procedures to dedicated interventionalists. Fellows can inquire about the amount of elective time (weeks, months), which subspecialties are available, and what type of studies or procedures they can perform while on these electives, and if there are any restrictions. Fellows can also inquire if they can use their elective time on more abdominal rotations, such as an MRI rotation. Different institutions may allow fellows to use the elective time for academic research, educational exhibits, quality improvement projects, or other scholarly pursuits. Lastly, fellows should be aware that electives may affect the amount of time they will be on dedicated abdominal rotations. While not all programs will have elective time, these rotations help supplement a fellow's education and prepare them for their next career.

### Workflow

Applicants should inquire about the day-to-day workflow on the rotations as this may affect their learning and study interpretations. Informative questions may include as follows: In a modality-based rotation, how are studies assigned? Do people read off a shared worklist across sites and what impact may that have on education? What is the role of the fellow? Are phone calls managed by the fellow or by a reading room coordinator? How do fellows share the responsibility of protocoling exams? Are readouts performed in person, remotely, or are preliminary interpretations assigned to attendings for review? What is the breakdown of studies a fellow may read on certain rotations in one day? Many of these questions are fair to ask during an interview, not only to faculty but to current and former fellows.

Given safety measures implemented due to the novel Coronavirus pandemic, institutions pivoted their workflow to adapt to recent challenges, changing from in-person to remote or virtual readouts with remote workstations for faculty, fellows, or both working at home [8]. Applicants may wish to ask programs how the pandemic has affected their operations and what they have done to maintain the quality of education and protect the safety of trainees and fellows. While working at home can have benefits of safety, limiting exposure to infections, doing so may impact other aspects of clinical care, including procedure time.

### Education and conferences

Fellowships should incorporate lectures to the curricula and integrate fellows into multidisciplinary conferences and tumor boards. These conferences are excellent resources to learn complex pathology, build rapport with referring providers, participate in patient management, and aiding in diagnosis of complex disease processes.

Didactic lectures introduce new and exciting topics in abdominal imaging, focusing on more advanced topics than those presented in residency. Applicants can inquire about the frequency of the lectures, as fellowships may offer lectures for portions of the year while others may offer these weekly year-round. Given the complexities of having rotations at multiple sites, virtual lecture presentations should be recorded so that fellows can review these at their leisure [8].

Fellows can also take part in multidisciplinary conferences and tumor boards. These meetings provide invaluable feedback from providers and help fellows understand the clinical impact of their interpretations. Fellows can expect to answer tough questions about the proper imaging follow-up or best exam to answer a clinical question. Each subspecialty conference includes a diverse treatment team of surgeons, oncologists, radiation oncologists, pathologists, and interventionalists and increases the visibility of the radiologist in the decision-making process and brings immense educational value [9]. Fellows can inquire which conferences are required and if they can optionally attend and present in other conferences to gain further expertise.

Other important meetings can include an interesting case conference series held daily, weekly, or periodically; medical student classes and workshops; and resident teaching during readouts and lectures, and journal clubs. Fellows may participate in Quality Improvement meetings to contribute to the section, improving workflow, increasing safety, and minimizing errors.

## Research

Depending on an applicant's interest, institutions may have a strong framework and resources for fellows to pursue research in their clinical fellowship year. Institutions with close collaborations between basic scientists, physicists, statisticians, and radiologists for large multi-centered studies and grants for basic science may appeal more to future clinician scientists. Other faculty members on a clinical service may have a strong background in clinical research. Fellows who are interested in an academic career may want to find mentors through imaging societies and research faculty profiles to reach out to members whose research closely matches their interests if they wish to continue or begin a new project at the start of the fellowship. For instance, if an applicant has interests in machine learning, they may wish to consider programs where the abdominal section has leaders in the informatics field to discuss potential opportunities for projects or collaboration.

Applicants can ask what resources a program may have to help fellows start a research project or other scholarly activity such as a quality improvement project. For instance, programs may gather a list of potential projects for fellows at the start of the year, while other times, fellows may ask the program director to help find a mentor to building upon a specific idea they wish to cultivate. Projects may include case-based image review, review articles, dictation templates, educational or scientific exhibits, protocol development, informatics, or quality improvement projects for safety, interpretations, or workflow [10]. Other quality improvement projects may include new fast MRI protocols, imaging specific organs, or developing a new test or template. Fellows should work with a faculty mentor to establish an outline and goals so that the project can be completed in one year.

Applicants can inquire about non-clinical academic time, which they can use towards their projects [10]. Fellowships may require a scholarly activity before issuing academic time and other programs may have limits to the frequency of non-clinical time. Availability of academic time can be subject to clinical need and satisfactory clinical performance of the fellow on rotations. Fellows may be able to use the academic time throughout the year to rotate on to other services, like elective time. Not all programs may give academic time as the focus of a clinical abdominal imaging fellowship is clinical work and education.

Some institutions include a two-year clinical and research fellowship in abdominal imaging, otherwise known as a NIH-Sponsored T32 Research Training Grant [11]. These programs are funded to promote physician-scientists pursuing a career in academic radiology. While these programs are beyond the scope of this article, interested applicants should research prospective institutions and mentors to find the program which best suits them.

## Jobs

For many fellows, the goal at the start of fellowship is to find a job. A fellow should consider the type of setting: academics or private practice. Academic careers can vary from research with basic science, clinical trials, or retrospective analyses to education and quality improvement. Fellows interested in this path should focus not only on their clinical training, but developing skills including but not limited to academic writing, project development, data analysis, process improvement, public speaking, and education. Some of these same qualities can be useful in private practice, excluding most large-scale research. Fellows opting for a career in private practice may want to dedicate their focus on choosing programs with higher clinical volumes to improve speed and efficiency while maintaining accuracy, however, this should be a requisite goal for a fellow in any practice setting.

Different fellowship programs may offer unique qualities that can help a fellow succeed in either setting. An institution with a greater research focus including sequence development, basic science, large grant funding, and clinical trials will often have more faculty that can mentor fellows for an academic career. Fellows may learn the nuances of setting up and implementing a research project, analyzing statistics or help with grant writing. Other institutions may have specialists in clinical education, working with fellows on educational exhibits, case reports, and review articles for those who favor training a new generation of radiologists, which can be found in either academics or private practice. Some institutions can help cultivate leaders in radiology, implementing clinical programs, quality improvement processes, business applications of radiology, or improving diversity and equity in healthcare, which are useful in any practice.

Each fellowship program has specific resources to help its trainees achieve the goal of obtaining a job. Programs may have an extensive alumni network across the country and supply a career night with panelists, discussing the types of jobs in radiology and giving tips to navigate the interview season. Applicants can inquire which resources are available to them to help them secure future employment. There are inherent advantages if the fellowship is near a specific region where the fellow seeks employment. In addition to cutting on transportation costs for interviews, institutions may have more local contacts as past graduates often return to their home state to find employment and settle down [12].

## Mentoring

Program directors are an invaluable resource for mentorship. As the advocate for fellows, the program director has experience in helping fellows navigate the interview process depending on the type of job. The program director helps develop the educational curricula and is open to feedback to make improvements not only for the current fellowship class but for future classes. Fellows should feel open to reach out with requests, ask for help, or inquire about areas of weakness. Program directors identify areas for improvement early on and develop a plan to help fellows improve over the course of the year. Applicants should ask about evaluations and feedback, as this is an essential part of self-learning [10]. Some programs will meet with fellows early in the year to inquire where they need to improve, what type of job they are looking for, setting goals and expectations, and formulating a plan to reach those goals.

Faculty also plays a key role in mentoring and serves as role models to fellows. Applicants should look to faculty members of a program whom they can self-identify. Many members of abdominal imaging sections play integral roles in diversity, inclusion, and equity initiatives, including women in radiology and under-represented minorities [13]. Other members may promote health and wellness in the department [14]. Lastly, faculty members may be a great resource to evaluate ideas for research or career advice. Applicants may want to consider these essential elements in mentoring when researching and deciding on a program.

## Time off requests

Applicants should consider the amount of vacation, meeting time, and leave offered to fellows. Most programs will have a similar number of vacation days to an ACGME-approved radiology residency. However, a significant amount of time away from work may be spent for interviews, which may require using those vacation days when seeking employment. Fellows may ask if the program supplies time off for interview. Programs that can provide vacation with fewer restrictions may be helpful for the interview season. Programs that allow vacation in 1- or 2-day increments rather than consecutive days or weeklong periods add flexibility. A larger fellowship class may potentially allow more flexibility in time off requests since there may be sufficient clinical coverage.

Fellows interested in taking time to present at a conference or attend a continuing medical education course can check with the program to see how many days are included for these activities.

An important but often overlooked request includes paid leave of absence. Different programs will have varying amounts of days for new parent leave, bereavement leave, and sick leave [15]. Furthermore, some institutions have implemented a COVID-19 leave in the wake of the pandemic to ease quarantining or help with seeking medical attention or childcare [16].

## Call structure

One of the most raised topics in fellowship interviews, the number and structure of calls is important in deciding a fellowship when other factors between programs are equal. Fellows should ask if a program has only call shifts, a night float, or both and the structure of these shifts, as this information may not be available on a program’s website [17]. While it may be difficult to understand the complexity of another institution's call structure, inquiring about what a fellow may need to read, how many studies they will be reading, and for how long a time can help evaluate the call burden. Fellows can ask about the overall number of days in a month or year when a fellow is on a call shift to weigh the call burden. Some call may affect your clinical rotations. Overnight or late night shifts and night floats can limit the amount of time and cases you see during the core rotations or electives during the day.

## Benefits

While education should be the main consideration in choosing a fellowship, benefits are an important part of acclimating to the rigors of training and wellness. Applicants should inquire about funds for licensing, moving, presentation at meetings, housing stipends, transportation benefits, and subsidized housing. Applicants looking to raise a family can ask current and former fellows how their experiences were during their fellowship year, what challenges they faced, and what programs were useful during the fellowship to accommodate their needs including childcare and medical appointments. Some programs have affiliated childcare facilities on campus or near campus housing, which may be a unique incentive for families deciding on a place to live. Programs may also provide lactation facilities for their trainees [18].

## Location and transportation

The transient nature of a 1-year fellowship presents an opportunity for applicants to consider living in a new location before settling into their next job. This can present challenges of moving twice within a brief period at the end of the fellowship. Applicants should consider the size of available space and the cost of housing near a hospital or campus. Communities where housing is affordable can supply ample space to raise a family or have pets. Programs in urban areas may have smaller apartment housing, but a stronger infrastructure for deliveries of food, groceries, and online orders with nearby restaurants and amenities without the need to own a car. Applicants may want to consider the importance of outdoor activities, with accessibility to nearby parks and recreational spaces for strolls, hiking, or exercise. Additional factors include how fellows will commute to work, if a car is necessary, or if they can use alternative modes of transportation including bicycles or buses. Urban communities often have ride-share and bike-share options for work, which may be subsidized for hospital employees. Some hospitals supply free accessible shuttles for employees or employee discount for parking and transportation costs.

## Summary

Choosing an abdominal imaging fellowship can be a challenging yet rewarding experience. Radiologists in training have this opportunity to explore the newest advances and exciting changes that abdominal radiology has to offer. Applicants will meet luminaries in the field and discuss their shared interests with faculty clinicians, educators, and researchers during the interview season. Applicants should consider key factors, including their desired career choices and location, as both may have a significant impact on the choice of programs. They should also use the interview to understand the section culture and day-to-day workflow, speaking not only with faculty, but current and former fellows to know how these programs best prepared them for their careers. Scheduling interviews at an applicant’s desired programs early in the season maximizes their chances for offers. And once an applicant accepts the offer and the interview season is complete, trainees can take their next big steps into a larger and expanding field of Abdominal Imaging.

## References

[1] “SCARD.” https://www.scardweb.org/about/policies (accessed Mar. 06, 2021).

[2] Magudia K (2020). Prospects of a Fellowship Match for Abdominal Imaging: A National Survey by the Society of Abdominal Radiology. J Am Coll Radiol.

[3] Hoffman DH, Rosenkrantz AB (2018). Defining the abdominal radiologist based on the current U.S. job market. Abdom. Radiol..

[4] Schwartz LH, Panicek DM, Berk AR, Li Y, Hricak H (2011). Improving communication of diagnostic radiology findings through structured reporting. Radiology.

[5] S. R. Baker, L. Luk, and K. Clarkin, “The Trouble With Fellowships,” *Journal of the American College of Radiology*, vol. 7, no. 6. Elsevier, pp. 446–451, Jun. 01, 2010, 10.1016/j.jacr.2010.01.020. 10.1016/j.jacr.2010.01.020 20522398

[6] Acgme, “ACGME Program Requirements for Graduate Medical Education in Abdominal Radiology.”

[7] C. M. Pfeifer, “The Fellowship Arms Race Revisited,” *Journal of the American College of Radiology*, vol. 13, no. 8. Elsevier B.V., pp. 891–892, Aug. 01, 2016, 10.1016/j.jacr.2016.05.017. 10.1016/j.jacr.2016.05.017 27493086

[8] Virarkar M, Jensen C, Javadi S, Saleh M, Bhosale PR (2020). Radiology Education Amid COVID-19 Pandemic and Possible Solutions. J. Comput. Assist. Tomogr..

[9] M. D. Lesslie and J. R. Parikh, “Multidisciplinary Tumor Boards: An Opportunity for Radiologists to Demonstrate Value,” *Academic Radiology*, vol. 24, no. 1. Elsevier USA, pp. 107–110, Jan. 01, 2017, 10.1016/j.acra.2016.09.006. 10.1016/j.acra.2016.09.006 27793581

[10] P. H. Chen and M. H. Scanlon, “Teaching Radiology Trainees From the Perspective of a Millennial,” *Academic Radiology*, vol. 25, no. 6. Elsevier USA, pp. 794–800, Jun. 01, 2018, 10.1016/j.acra.2018.02.008. 10.1016/j.acra.2018.02.008 29573938

[11] “Program Details | Research Training and Career Development.” https://researchtraining.nih.gov/programs/research-education/T32 (accessed Mar. 06, 2021).

[12] Mok PS, Probyn L, Finlay K (2016). Factors Influencing Radiology Residents’ Fellowship Training and Practice Preferences in Canada. Can. Assoc. Radiol. J..

[13] E. M. Webb, M. D. Bucknor, and D. M. Naeger, “Diversity and Inclusion: Now Radiology Must Walk the Walk,” *Journal of the American College of Radiology*, vol. 15, no. 4. Elsevier B.V., pp. 687–688, Apr. 01, 2018, 10.1016/j.jacr.2017.10.038. 10.1016/j.jacr.2017.10.038 29242022

[14] Porrino J, Mulcahy MJ, Mulcahy H, Relyea-Chew A, Chew FS (2017). Emotional Wellness of Current Musculoskeletal Radiology Fellows. Acad. Radiol..

[15] F. Ghazi Sherbaf, D. D. M. Lin, and D. M. Yousem, “Parental Leave Policy in Radiology Residency Programs: Current Status,” *J. Am. Coll. Radiol.*, vol. 17, no. 9, pp. 1163–1171, Sep. 2020, 10.1016/j.jacr.2019.12.032. 10.1016/j.jacr.2019.12.032 32275902

[16] M. D. Alvin, E. George, F. Deng, S. Warhadpande, and S. I. Lee, “The Impact of COVID-19 on Radiology Trainees,” *Radiology*, vol. 296, no. 2. Radiological Society of North America Inc., pp. 246–248, Aug. 01, 2020, 10.1148/radiol.2020201222. 10.1148/radiol.2020201222 PMC7233407 32216719

[17] Ruddell JH, Hartley-Blossom ZJ, Bajaj AI, Grand D, Eltorai AEM (2019). Analysis of Abdominal Radiology fellowship website content and comprehensiveness. Abdom. Radiol..

[18] E. K. Arleo, J. R. Fielding, J. B. Lightfoote, W. Shields, E. I. Bluth, and K. J. Macura, “Radiology and Radiation Oncology Practices Should Provide Lactation Facilities for All Eligible Employees,” *Journal of the American College of Radiology*, vol. 12, no. 10. Elsevier, pp. 1127–1128, 2015, 10.1016/j.jacr.2015.07.036. 10.1016/j.jacr.2015.07.036 26435125

